# Incidence and risk factors of incisional hernia after CRS and HIPEC for colorectal-appendiceal peritoneal malignancies: a retrospective cohort study

**DOI:** 10.1186/s12893-026-04106-5

**Published:** 2026-07-31

**Authors:** Vural Argın, Ahmet Orhan Sunar, Nurdan Bezir, Muhammed İkbal Akın, Ömer Faruk Kandaz, İsmail Ertuğrul, Erdal Polat, Adnan Gündoğdu, Mustafa Duman

**Affiliations:** 1 Department of General Surgery, Marmara University Pendik Training and Research Hospital, Istanbul, Türkiye Turkey; 2 Department of Gastrointestinal Surgery, University of Health Sciences, Kartal Koşuyolu Yüksek İhtisas Training and Research Hospital, Istanbul, Türkiye Turkey; 3 Department of General Surgery, Kartal Dr. Lütfi Kırdar City Hospital, Istanbul, Türkiye Turkey; 4 Department of General Surgery, Sancaktepe Şehit Prof. Dr. İlhan Varank Training and Research Hospital, Istanbul, Türkiye Turkey

**Keywords:** Colorectal cancer, Pseudomyxoma peritonei, Cytoreductive surgery, Hyperthermic intraperitoneal chemotherapy, Incisional hernia, Risk factors

## Abstract

**Background:**

Incisional hernia is a common late complication after extensive abdominal surgery and may significantly impair quality of life. The incidence and risk factors of incisional hernia in patients undergoing cytoreductive surgery (CRS) combined with hyperthermic intraperitoneal chemotherapy (HIPEC) for peritoneal surface malignancies of colorectal or appendiceal origin remain insufficiently studied. This study aimed to evaluate the incidence of incisional hernia and to identify factors associated with incisional hernia in this specific patient population.

**Methods:**

This retrospective, single-center cohort study included 147 patients who underwent CRS+HIPEC between January 2018 and January 2024. Demographic data, tumor characteristics, surgical details, and comorbidities were obtained from a prospectively maintained database. All patients underwent midline laparotomy, and HIPEC was performed using either mitomycin C or oxaliplatin with 5-fluorouracil/leucovorin via the closed technique at 42–43 °C for 30–90 min. Patients were followed for at least 18 months, and incisional hernia was diagnosed by physical examination and/or radiologic imaging. Univariable and multivariable logistic regression analyses were used to identify associated factors.

**Results:**

The mean age was 54.07 ± 11.70 years, with an equal sex distribution, and the mean follow-up was 30.7 ± 16.2 months. During follow-up, 83 patients (56.5%) developed an incisional hernia. Prior open abdominal surgery (OR 2.621; 95% CI 1.155–5.946; *p* = 0.021) and a history of hernia (OR 13.682; 95% CI 2.928–63.941; *p* < 0.001) were significant predictors in univariable analysis and remained independently associated with incisional hernia in multivariable analysis.

**Conclusions:**

The incidence of incisional hernia after CRS+HIPEC is high, and patients with previous open abdominal surgery or preexisting hernia should be considered at increased risk during preoperative assessment and postoperative follow-up.

## Background

Colorectal cancer (CRC) is among the most common malignancies worldwide and represents one of the leading causes of cancer-related mortality [1]. Synchronous peritoneal metastases are present in approximately 10–15% of patients with colorectal cancer at the time of diagnosis, whereas metachronous peritoneal metastases develop in approximately 15–30% of patients during follow-up [2, 3]. In this subgroup, survival outcomes remain poor despite systemic chemotherapy. While patients receiving chemotherapy alone have a median survival of approximately 16.3 months, selected patients undergoing cytoreductive surgery (CRS) combined with hyperthermic intraperitoneal chemotherapy (HIPEC) have been reported to achieve survival durations of up to 40 months [4]. In recent years, CRS in combination with HIPEC has been incorporated into standard treatment strategies for appropriately selected patients, given its potential to confer long-term survival benefits [5].

CRS+HIPEC is a complex surgical procedure associated with substantial perioperative morbidity. However, advances in patient selection, perioperative management, and increasing surgical experience have significantly improved outcomes. In experienced centers that have surpassed the learning curve, severe postoperative morbidity has been reported to remain below 20%, with morbidity and mortality rates comparable to those of other major abdominal procedures, such as pancreaticoduodenectomy (Whipple procedure) [6, 7]. Postoperative complications continue to significantly influence the clinical course of these patients, both in the short and long term. Although overall postoperative complication rates reported in the literature vary widely (14–70%), depending on patient selection, disease burden, and institutional experience, incisional hernia is particularly relevant, as it frequently occurs following extensive midline laparotomies and represents a late complication that substantially impairs quality of life [8, 9]. Beyond increasing morbidity, incisional hernia can lead to chronic pain, cosmetic deformity, and potentially life-threatening conditions such as bowel obstruction or strangulation [10].

In the general population, the incidence of incisional hernia after major abdominal surgery has been reported to range from 10% to 20% [11]. Identified risk factors include surgical site infection, obesity, malnutrition, advanced age, immunosuppression, smoking, and technical aspects of fascial closure [12]. In patients undergoing CRS+HIPEC, however, the prolonged operative time, the extent of peritoneal surface resection, the potential detrimental effects of hyperthermic chemotherapy on tissue healing, and the presence of additional comorbidities may further increase the risk of incisional hernia. Nevertheless, there is limited evidence in the literature regarding the incidence and associated risk factors of incisional hernia in this specific patient population.

The present study aimed to evaluate the incidence of incisional hernia and to identify contributing risk factors in patients with peritoneal surface malignancies of colorectal or appendiceal origin who underwent CRS+HIPEC.

## Methods

### Study design and patient selection

This retrospective, single-center cohort study included patients aged 18–75 years who underwent curative-intent (CC-0 or CC-1) cytoreductive surgery combined with hyperthermic intraperitoneal chemotherapy (HIPEC) for peritoneal surface malignancies of colorectal or appendiceal origin between January 2018 and January 2024. Patients diagnosed with pseudomyxoma peritonei were of appendiceal origin and confirmed histopathologically. Patients who underwent only diagnostic laparoscopy or who required mesh reconstruction following abdominal wall resection were excluded. The institutional CRS+HIPEC program was initiated in 2018 by two senior surgeons. During this period, a multidisciplinary team was established, patient selection criteria were standardized, surgical protocols were unified, HIPEC procedures were structured, and perioperative safety algorithms were implemented according to the recommendations of the 2008 Peritoneal Surface Oncology Group International (PSOGI) meeting [13]. This study was approved by the Institutional Ethics Committee (Approval No. 2025/11/1181), and the requirement for informed consent was waived due to the retrospective design. Further details are provided in the Declarations section.

### Data collection and variables

Patient data were prospectively collected using a dedicated institutional database. The recorded variables included demographic characteristics (age, sex, and body mass index), tumor histology, presence of diabetes, history of hernia, peritoneal cancer index (PCI), smoking history, and preoperative imaging findings. The PCI was calculated intraoperatively according to the Sugarbaker classification system, and completeness of cytoreduction was assessed using the Completeness of Cytoreduction (CC) score, where CC-0 indicated no visible residual disease and CC-1 indicated residual tumor nodules ≤ 2.5 mm [13].

### Follow-up and definition of incisional hernia

Patients were followed for at least 18 months postoperatively. Follow-up consisted of clinical examinations and radiological assessments (computed tomography and/or ultrasonography) to evaluate disease recurrence and the development of incisional hernia. Patients were followed at regular intervals throughout the follow-up period. Although the exact timing of hernia development could not be precisely determined, the follow-up period at which the hernia was first detected was recorded. Incisional hernia was defined as any postoperative abdominal wall defect identified based on clinical findings, including physical examination and/or patient-reported symptoms, and subsequently confirmed by imaging. Therefore, all hernias included in the study were considered clinically significant, and no cases were diagnosed solely based on imaging findings. Prior incisional hernia was defined as a documented history of abdominal wall hernia diagnosed before CRS+HIPEC, regardless of whether previous hernia repair had been performed.

### Surgical technique

All patients received deep vein thrombosis prophylaxis with low-molecular-weight heparin and anti-embolic stockings. A urinary catheter was placed in all cases. Procedures were performed in the Lloyd-Davies position. A midline incision extending from the xiphoid process to the symphysis pubis was used, and previous incision scars were excised due to the potential risk of tumor implantation. Routine resection of the umbilicus was not performed during CRS+HIPEC procedures. Given the standardized use of a midline incision in all patients, all incisional hernias observed during follow-up were located along the midline. Intraoperative disease burden was assessed using the Peritoneal Cancer Index as described by Sugarbaker. Disease-specific peritonectomy procedures were performed for pseudomyxoma peritonei and colorectal cancer. Depending on disease extent, procedures included greater omentectomy with resection of the omental bursa (lesser sac), left lateral parietal peritonectomy, right parietal peritonectomy ± right colon resection, pelvic peritonectomy ± sigmoid colon resection ± hysterosalpingo-oophorectomy, lesser omentectomy ± antrectomy ± cholecystectomy, right upper quadrant peritonectomy ± Glisson capsule resection, left upper quadrant peritonectomy ± splenectomy, and additional bowel resections. Optimal CRS was defined as a completeness of cytoreduction (CC) score of 0 or 1.

### HIPEC protocol

Following CRS, HIPEC was performed using a closed technique with a perfusion machine. The chemotherapeutic regimens included intraperitoneal oxaliplatin or intraperitoneal mitomycin C. In patients undergoing oxaliplatin-based HIPEC, 5-fluorouracil and leucovorin were administered intravenously simultaneously according to the standard oxaliplatin-based HIPEC protocol. After achieving an intra-abdominal temperature of 42 °C, perfusion continued for 30–90 min while maintaining a temperature range between 42 °C and 43 °C. Chemotherapy was circulated through five separate drains at a flow rate of 1000 cc/min. Intestinal anastomoses were performed after completion of HIPEC. In patients without anastomosis, definitive abdominal closure was performed before HIPEC.

### Abdominal wall closure technique

The abdominal wall was closed using a standardized mass-closure technique [14]. The fascia was approximated with No. 1 loop polydioxanone (PDS) suture in a continuous running fashion, taking 1-cm bite intervals and 1-cm edge distance, ensuring uniform tension along the suture line. The subcutaneous tissue was irrigated, and skin closure was completed using staples or absorbable sutures at the surgeon’s discretion. This standardized technique was routinely used in our center throughout the study period to maintain consistency in abdominal wall closure among patients undergoing CRS+HIPEC procedures.

### Statistical analysis

All statistical analyses were performed using IBM SPSS Statistics version 26.0 (IBM Corp., Armonk, NY, USA). Continuous variables were expressed as mean ± standard deviation, and categorical variables as frequencies and percentages. Group comparisons were conducted using the chi-square test or Fisher’s exact test for categorical variables. For continuous variables, the Student’s t-test was used when parametric assumptions were met; otherwise, the Mann–Whitney U test was applied. Univariate analyses were first conducted to identify factors associated with incisional hernia. Multivariable logistic regression analysis was performed to determine independent risk factors, including variables that were either statistically significant (*p* < 0.20) in univariable analysis or considered clinically relevant based on existing literature. Odds ratios (ORs) and 95% confidence intervals (CIs) were calculated. A p-value < 0.05 was considered statistically significant.

## Results

A total of 147 patients who underwent cytoreductive surgery combined with hyperthermic intraperitoneal chemotherapy were included in the study. The mean age was 54.07 ± 11.70 years, with 50.3% female (*n* = 74) and 49.7% male (*n* = 73) patients. The mean follow-up time was 30.7 ± 16.2 months, and the median follow-up was 26.3 months (range 18–69 months). Of the patients, 108 (73.5%) had colorectal tumors as the primary diagnosis, while 39 (26.5%) had pseudomyxoma peritonei (PMP). The distribution of body mass index (BMI) was as follows: ≥30 in 65 patients (44.2%), 25–30 in 39 patients (26.5%), and < 25 in 43 patients (29.2%). Among comorbidities, diabetes mellitus was present in 16 patients (10.9%), hypertension in 36 (24.5%), ischemic heart disease in 7 (4.8%), and chronic obstructive pulmonary disease (COPD) in 4 (2.7%). A history of smoking was identified in 10 patients (6.8%). The PCI was < 10 in 101 patients (68.7%) and ≥ 10 in 46 patients (31.3%). In addition, 68 patients (46.3%) had a history of previous open abdominal surgery, and 38 (25.9%) had a history of incisional hernia (Table 1).

**Table 1:** Baseline demographic and clinical characteristics of the study population

Variable	Value
Age (years), mean ± SD	54.07 ± 11.70
Sex, *n* (%)	
Female	74 (50.3)
Male	73 (49.7)
Primary tumour, *n* (%)	
Colon + rectum	108 (73.5)
﻿Pseudomyxoma peritonei (PMP)	39 (26.5)
Body mass index (BMI), *n* (%)	
<25	43 (29.2)
25–29.9	39 (26.5)
≥30	65 (44.2)
Comorbidities, *n* (%)	
Diabetes	16 (10.9)
Hypertension	36 (24.5)
Ischemic heart disease	7 (4.8)
Chronic obstructive pulmonary disease (COPD)	4 (2.7)
Tobacco use, *n* (%)	
Yes	10 (6.8)
No	137 (93.2)
Length of cytoreductive surgery (hours), mean ± SD	7.06 ± 3.00
Peritoneal Cancer Index, *n* (%)	
<10	101 (68.7)
≥10	46 (31.3)
Prior open abdominal surgery, *n* (%)	
Yes	68 (46.3)
No	79 (53.7)
Prior incisional hernia, *n* (%)	
Yes	38 (25.9)
No	109 (74.1)
Incisional hernia, *n* (%)	
Yes	83 (56.5)
No	64 (43.5)
Mean follow-up duration (months), mean ± SD (range)	30.7 ± 16.2 (18–69)

*SD* Standard deviation, *BMI* Body mass index, *COPD* Chronic obstructive pulmonary disease, *PMP* Pseudomyxoma peritonei, *PCI* Peritoneal cancer index

The mean duration of CRS was 7.06 ± 3.00 h. While colectomy was performed in all cases, additional resections were also carried out: peritonectomy in 82 patients (55.7%), small bowel resection in 37 (25.1%), splenectomy in 35 (23.8%), hepatectomy in 12 (8.1%), gastrectomy in 6 (4.1%), and hysterectomy in 30 (20.4%). A total of 11 patients (7.4%) underwent end colostomy, and 37 patients (25.1%) had a protective ileostomy. The most frequently used HIPEC regimen was oxaliplatin combined with 5-FU/leucovorin in 133 patients (90.4%), followed by mitomycin C in 10 patients (6.8%) (Table 2).

**Table 2:** Operative details and surgical procedures of the study population

Operative data	*N* = 147
Length of cytoreductive surgery (hours), mean ± SD	7.06 ± 3.00
Chemotherapeutic agents, *n* (%)	
Oxaliplatin + 5-FU/leucovorin	133 (90.4)
Mitomycin C	10 (6.8)
Mitomycin C + cisplatin	2 (1.4)
Cisplatin	1 (0.7)
Doxorubicin + cisplatin	1 (0.7)
Small bowel resection, *n* (%)	37 (25.1)
Gastrectomy, *n* (%)	6 (4.1)
Colectomy, *n* (%)	147 (100)
Splenectomy, *n* (%)	35 (23.8)
Hepatectomy, *n* (%)	12 (8.1)
Hysterectomy, *n* (%)	30 (20.4)
Adrenalectomy, *n* (%)	1 (0.7)
Peritonectomy, *n* (%)	82 (55.7)
Colostomy, *n* (%)	11 (7.4)
Ileostomy, *n* (%)	37 (25.1)

5-FU 5-fluorouracil

During follow-up, 83 patients (56.5%) developed an incisional hernia. All hernias were located along the midline incision. In univariate analysis, previous open abdominal surgery (*p* < 0.001) and a prior history of hernia (*p* < 0.001) were significantly associated with the development of incisional hernia. No significant associations were observed with age, sex, BMI, diabetes, COPD, smoking status, PCI, wound infection, or peritonectomy. In multivariable logistic regression analysis, previous open abdominal surgery (OR 2.621; 95% CI 1.155–5.946; *p* = 0.021) and a history of prior hernia (OR 13.682; 95% CI 2.928–63.941; *p* < 0.001) remained independently associated with incisional hernia (Tables 3 and 4).

**Table 3:** Univariate analysis of factors associated with incisional hernia

Variable	Hernia – Yes, *n* (%)	Hernia – No, *n* (%)	*p*-value
Age (years)			
18–49	26 (31.3)	18 (28.1)	
50–64	39 (47.0)	36 (56.3)	
≥65	18 (21.7)	10 (15.6)	0.490
Sex			
Female	40 (48.2)	34 (53.1)	
Male	43 (51.8)	30 (46.9)	0.553
Body mass index (BMI)			
<25	22 (26.5)	21 (32.8)	
25–29.9	22 (26.5)	17 (26.5)	
≥30	39 (47.0)	26 (40.7)	0.685
Length of cytoreductive surgery (hours)			
<6	33 (39.8)	19 (29.7)	
≥6	50 (60.2)	45 (70.3)	0.205
Prior open abdominal surgery			
Yes	53 (63.9)	15 (23.4)	
No	30 (36.1)	49 (76.6)	< 0.001
Diabetes			
Yes	8 (9.6)	8 (12.5)	
No	75 (90.4)	56 (87.5)	0.581
Peritonectomy			
Yes	47 (56.6)	35 (54.7)	
No	36 (43.4)	29 (45.3)	0.814
Chronic obstructive pulmonary disease (COPD)			
Yes	1 (1.2)	3 (4.7)	
No	82 (98.8)	61 (95.3)	0.198
Tobacco use			
Yes	5 (6.0)	5 (7.8)	
No	78 (94.0)	59 (92.2)	0.669
Prior incisional hernia			
Yes	36 (43.4)	2 (3.1)	
No	47 (56.6)	62 (96.9)	< 0.001
Peritoneal Cancer Index			
<10	57 (68.7)	44 (68.8)	
≥10	26 (31.3)	20 (31.2)	0.992
Surgical wound infection			
Deep	18 (21.7)	17 (26.6)	
Superficial	7 (8.4)	4 (6.3)	
No	58 (69.9)	43 (67.2)	0.629

*BMI* Body mass index, *COPD* Chronic obstructive pulmonary disease

**Table 4:** Multivariable analysis of factors associated with incisional hernia

Variable	OR	95% CI	*p*-value
Age (years)			
50–64 vs. 18–49	1.25	0.468–3.317	0.660
≥65 vs. 18–49	1.66	0.678–4.070	0.267
Body mass index (BMI)			
25–29.9 vs. < 25	1.128	0.472–2.695	0.787
≥30 vs. < 25	0.824	0.367–1.850	0.638
Length of cytoreductive surgery ≥ 6 h vs. < 6 h	0.640	0.320–1.280	0.207
Prior open abdominal surgery (Yes vs. No)	2.621	1.155–5.946	0.021
Prior incisional hernia (Yes vs. No)	13.682	2.928–63.941	< 0.001
Peritoneal Cancer Index ≥ 10 vs. < 10	0.997	0.493–2.013	0.992
Wound infection (Yes vs. No)	1.133	0.562–2.285	0.727
Diabetes (Yes vs. No)	1.339	0.474–3.787	0.582
COPD (Yes vs. No)	4.033	0.409–39.716	0.232

Reference categories: age 18–49, BMI < 25, surgery < 6 h, no prior open abdominal surgery, no prior incisional hernia, PCI < 10, no wound infection, no diabetes, no COPD

*OR* Odds ratio, *CI* Confidence interval, *BMI* Body mass index, *COPD* Chronic obstructive pulmonary disease

## Discussion

In this retrospective study, the development of incisional hernias and factors associated with incisional hernia were examined in patients who underwent CRS+HIPEC for peritoneal surface malignancies of colorectal or appendiceal origin. The analysis showed that previous open abdominal surgery and a history of hernia were independently associated with incisional hernia. In contrast, demographic characteristics, metabolic conditions, disease burden, and the extent of surgical resection did not show a significant association with the development of incisional hernia.

CRS+HIPEC is a complex intervention associated with significant morbidity due to prolonged operative time and extensive peritoneal dissection [15]. Moreover, hyperthermic intraperitoneal chemotherapy has been reported to adversely affect tissue healing [16]. Among the late postoperative complications following HIPEC, incisional hernia represents a clinically important issue [17]. In the literature, the incidence of incisional hernia after CRS+HIPEC ranges from 7% to 17%, with advanced age, female sex, obesity, and previous abdominal surgery identified as independent risk factors [18]. In our cohort, however, the incidence of incisional hernia was considerably higher at 56.5%. Prior open abdominal surgery and a history of incisional hernia were independently associated with incisional hernia in our analysis. These findings suggest an association between previous abdominal surgery and the occurrence of incisional hernia; however, the underlying biological mechanisms were not directly evaluated in the present study. The relatively wide confidence interval observed for prior abdominal surgery suggests that this variable may not represent a direct causal factor alone and may also reflect other underlying risk conditions. Previous laparotomies may be associated with impaired abdominal wall integrity, repeated surgical trauma, adhesions, altered tissue perfusion, or more complex disease burden, all of which could contribute to fascial failure following extensive CRS+HIPEC procedures. Therefore, this association should be interpreted cautiously, particularly considering the retrospective study design and the relatively limited sample size. Furthermore, the extensive and traumatic nature of CRS+HIPEC may also be associated with a higher incidence of incisional hernia, particularly in high-risk patient populations. In addition, the abdominal closure technique used in our center, which differs from the small-bite approach, may have contributed to the elevated incidence observed, although this factor was not directly evaluated in the present study [19, 20]. Therefore, preoperative assessment of patients scheduled for CRS+HIPEC should carefully consider prior abdominal surgery and hernia history. Whether alternative closure techniques may reduce hernia rates in this population warrants further investigation.

In our analysis, no significant association was observed between incisional hernia and variables such as age, sex, body mass index, diabetes, smoking, peritoneal cancer index, and wound infection. However, the literature reports that some of these factors may play an important role. Itatsu et al. reported a significantly higher incidence of incisional hernia in patients with a BMI ≥ 25 [19]. Similarly, smoking has been shown in various series to impair wound healing and significantly increase the risk of incisional hernia [21]. Diabetes mellitus has also been identified as a factor predisposing to wound complications and has been associated with a significantly increased risk of incisional hernia [22]. Wound infection has consistently been reported as one of the factors most strongly associated with incisional hernia, with some series reporting an incidence of incisional hernia as high as 30% in patients who developed surgical site infections [23]. The lack of significant associations with these variables in our study may be related to the relatively limited sample size, the predominance of other strongly associated factors in our cohort, particularly previous laparotomy and a history of hernia, or differences in follow-up protocols.

Prolonged operative time is considered a potential mechanism associated with the development of incisional hernia. In lengthy surgeries, tissue hypoxia, impaired perfusion, and an increased inflammatory response may adversely affect wound healing, leading to weakening of the fascial integrity [19, 24]. Similarly, extensive surgical procedures such as peritonectomy may further impair fascial healing because of greater tissue trauma. Elias et al. reported that multiple peritoneal resections increase the risk of late postoperative complications [25], while Piehler et al. demonstrated that hyperthermic chemotherapy impairs fibroblast function and reduces collagen synthesis [26]. In our series, the mean operative time was 7.06 ± 3.00 h, and 56.6% of patients who developed an incisional hernia had undergone peritonectomy. However, neither prolonged operative duration nor peritonectomy was significantly associated with the development of incisional hernia in our cohort. In univariate analysis, operative duration was not significantly associated with incisional hernia (*p* = 0.205), and multivariate logistic regression likewise showed no independent association (OR 0.640; 95% CI 0.320–1.280; *p* = 0.207). Likewise, although a greater proportion of patients with incisional hernia had undergone peritonectomy, this difference did not reach statistical significance. These findings suggest that operative duration and peritonectomy were not independent risk factors in the present study. The absence of a significant association may be explained by the relatively limited sample size and the stronger influence of established predictors, particularly previous open surgery and a history of hernia. Nevertheless, given the biological plausibility and previous literature, the potential contribution of extensive surgical procedures to impaired fascial healing warrants further investigation in larger prospective studies.

From a clinical perspective, the extensive and complex nature of CRS+HIPEC procedures, including prolonged operative duration, wide abdominal dissections, multivisceral resections, peritonectomy procedures, and the potential adverse effects of hyperthermic intraperitoneal chemotherapy on fascial healing, has been proposed to contribute to postoperative incisional hernia in previous studies. However, in our cohort, only previous open abdominal surgery and a history of incisional hernia were independently associated with incisional hernia. Therefore, these findings may help identify patients at increased risk of incisional hernia who could benefit from preventive strategies during abdominal wall closure. In selected patients with a history of prior laparotomy or incisional hernia, alternative closure techniques, abdominal wall reinforcement methods, or prophylactic mesh applications may be considered to reduce the risk of postoperative incisional hernia. Further prospective studies are needed to determine the optimal preventive approach in patients undergoing CRS+HIPEC.

### Limitations

This study has several limitations. First, its retrospective and single-center design may introduce selection bias and potential gaps in data collection. Second, the sample size is relatively limited, and particularly in subgroups (e.g., patients receiving mitomycin), small numbers may have reduced the statistical power. Additionally, although the diagnosis of incisional hernia was primarily based on clinical examination and imaging, a standardized protocol was not applied to all patients, which may have led to under- or overestimation of the true incidence. Moreover, although all hernias included in the study were considered clinically relevant and were not diagnosed solely based on imaging findings, symptom severity and the need for surgical repair were not analyzed separately. Therefore, minor symptomatic defects and more advanced hernias requiring intervention were evaluated within the same outcome category. Furthermore, previous abdominal surgery was recorded only as a binary variable (yes/no), and the Prior Surgical Score (PSS) described by Sugarbaker could not be assessed retrospectively because the detailed information required for its calculation was not consistently available. Therefore, the extent and complexity of previous abdominal surgery could not be evaluated in the present study. Additionally, in patients with a prior hernia history, the postoperative hernia may represent a recurrence rather than a de novo defect, and this distinction could not be made retrospectively. Because incisional hernia is a time-dependent outcome, variations in follow-up duration among patients may also have influenced hernia detection rates. Furthermore, factors such as variations in surgical technique, suture materials, and closure methods could not be analyzed in detail. Finally, although the median follow-up was 26 months, some patients had shorter observation periods, potentially limiting the assessment of long-term outcomes. Despite these limitations, our study contributes a relatively large series to the literature by providing data on the incidence and factors independently associated with incisional hernia in patients undergoing CRS+HIPEC, a population for which limited evidence currently exists.

## Conclusions

Incisional hernia is a common postoperative complication in patients undergoing CRS+HIPEC, emphasizing the need for careful assessment of individual risk profiles. Our findings indicate that a history of prior open abdominal surgery and the presence of a previous hernia were independently associated with incisional hernia in this setting. In contrast, demographic characteristics, comorbidities, and disease-related variables were not significantly associated with incisional hernia in our cohort. These results underscore the importance of preoperative risk assessment and personalized postoperative monitoring strategies to reduce hernia-related morbidity.

## Data Availability

The datasets used and/or analysed during the current study are available from the corresponding author on reasonable request.

## Abbreviations

BMI: Body mass index
CC: Completeness of cytoreduction
CI: Confidence interval
COPD: Chronic obstructive pulmonary disease
CRC: Colorectal cancer
CRS: Cytoreductive surgery
5-FU: 5-fluorouracil
HIPEC: Hyperthermic intraperitoneal chemotherapy
OR: Odds ratio
PCI: Peritoneal cancer index
PDS: Polydioxanone
PMP: Pseudomyxoma peritonei
PSOGI: Peritoneal Surface Oncology Group International
PSS: Prior Surgical Score
